# Using 3D printing to fabricate realistic test projectiles for natural fragmentation from buried charges

**DOI:** 10.1007/s43939-020-00004-6

**Published:** 2021-01-11

**Authors:** K. J. Hodder, F. Coghe, G. Kechagiadakis, R. J. Chalaturnyk

**Affiliations:** 1 Department of Chemical and Materials Engineering, Donadeo Innovation Centre for Engineering, University of Alberta, 9211-116 Street NW, Edmonton, AB T6G 1H9 Canada; 2 Department of Weapon Systems and Ballistics, Royal Military Academy, Renaissance Avenue 30, 1000 Brussels, Belgium; 3 Department of Civil and Environmental Engineering, Donadeo Innovation Centre for Engineering, University of Alberta, 9211-116 Street NW, Edmonton, AB T6G 1H9 Canada

## Abstract

Buried charges such as improvised explosive devices continue to be one of the most lethal and hidden threats service members face. On detonation, ground debris near the blast area is accelerated towards service members as secondary fragmentation, consisting of sand, gravel and rocks. In order to mitigate injury, protective equipment can be worn, yet it is difficult to gather accurate data for engineering decisions when the standard test uses a fragment simulating projectile made from metal. It is difficult to test secondary fragmentation from ground debris due to the natural heterogeneity and variance of the material. A methodical and reproducible method of testing fragmentation damage from ground debris was developed to study and improve protective equipment against natural secondary fragmentation. We present herein the novel process of 3D-printing ballistic projectiles from silica sand, followed by launching with an air canon. Outlined within are the successes, challenges and proposed implementations of the technology. The 3D-printed sand projectiles achieved speeds over 170 m/s, resulting in measurable damage to single Kevlar sheets. Other flight parameters such as yaw and rotation were captured, resulting in observations about design and shape of the projectiles. It was found that one design performed better in terms of velocity, rotation and impact. The technology has the potential to disrupt the protective equipment sector by providing a controlled means of assessing natural fragmentation damage.

## Introduction

Fragmentation damage is a serious concern in combat and peacekeeping missions as it has grown in popularity in modern combat [1]. In conflict, buried charges such as Improvised Explosive Devices (IEDs) are a hidden and lethal threat that can be crafted from household items. To design personal protective equipment (PPE) or armour systems against buried charges, Fragment Simulating Projectiles (FSPs) are fabricated from metal and launched at high speeds to simulate fragmentation damage in a laboratory setting. The US military specifies that cold-rolled, annealed steel shall be used to manufacture FSPs with specific diameters to represent 0.22 Caliber (5.6 mm), 0.33 Caliber (8.4 mm), 0.5 Caliber (12.7 mm), and 20 mm projectiles [2]. Although attempts have been made to standardize fragmentation testing by defining standard alloys and diameters, almost all FSPs are made from metal [3–6]. These metallic FSPs do not replicate the fragmentation that is accelerated from a buried IED. When an IED is buried, the ground material and strata directly above the device is accelerated due to the blast wave and directed at active service members. Since there is no current projectile fabricated from ground material, there is a severe mismatch between laboratory testing and active duty in terms of fragmentation damage. Thus, engineering decisions toward protective equipment worn by service members cannot be made accurately. The uncertainty results in comfort and fit being sacrificed for extra protection. However, discomfort and reduced mobility can pose an even greater danger to service members. When uncomfortable or cumbersome, service members either neglect the equipment or are hampered by extra weight in service [7]. A widely-used method to characterize the damage from natural fragmentation is an arena type experiment, where ground material is loaded into a canister and detonated. PPE is lined in a circle around the explosion in an attempt to “catch” any fragmentation. However, the damage caused is variable [8]. There is no agreed upon standard test, as any proposal has inherent problems with repeatability due to the natural heterogeneity of ground material in terms of shape, intensity of damage, as well as mechanical and flight trajectory [8–11].

In order to address the issue of an inaccurate projectile for natural fragmentation testing, additive manufacturing (AM) or 3D-printing (3DP) has been shown to be probable solution. More specifically, binder jetting AM was used to combine silica sand and a polymer binder to successfully fabricate 3D-printed rock that challenges traditional thinking about synthetic rock. Binder jetting AM is a form of 3DP that utilizes a print head to jet a liquid binder onto a bed of powder, layer by layer, until the part is completed. The mechanical [12–16] and hydraulic [17, 18] properties of 3D-printed rock have been quantified and compared to natural sandstone. The strongest critique of 3D-printed rock was that the unconfined compressive strength was not comparable to the strength of natural rock. However, a pre-treatment method has been developed involving silane coupling agents to increase the bond strength between particles, which increases the overall strength of 3D-printed rock [19]. More recently, the mean effects that individual printing parameters have on the strength and density of 3D-printed rock has been explored. For example, it has been shown that the volume fraction of binder has a significant effect on the overall compressive strength and density of 3D-printed rock [13, 16]. Additionally, a recent paper that was submitted shows that the amount of heat and travel speed of the hopper during printing changes the density of 3D-printed specimens.

There is a critical scientific knowledge gap between ballistic testing, FSPs and body armour, which after decades of testing remains unsolved. Since there is no projectile that is fabricated from ground material in a repeatable, reproducible fashion, natural fragmentation damage testing between a laboratory and active duty is ignored or accepted. Herein we have successfully fabricated samples from ground material such as silica sand that can be fabricated into any shape via 3D-printing. The 3D-printed sand projectiles within challenge traditional thinking of natural fragmentation damage testing.

## Experimental procedure

### 3D printing of samples

**Fig. 1:**
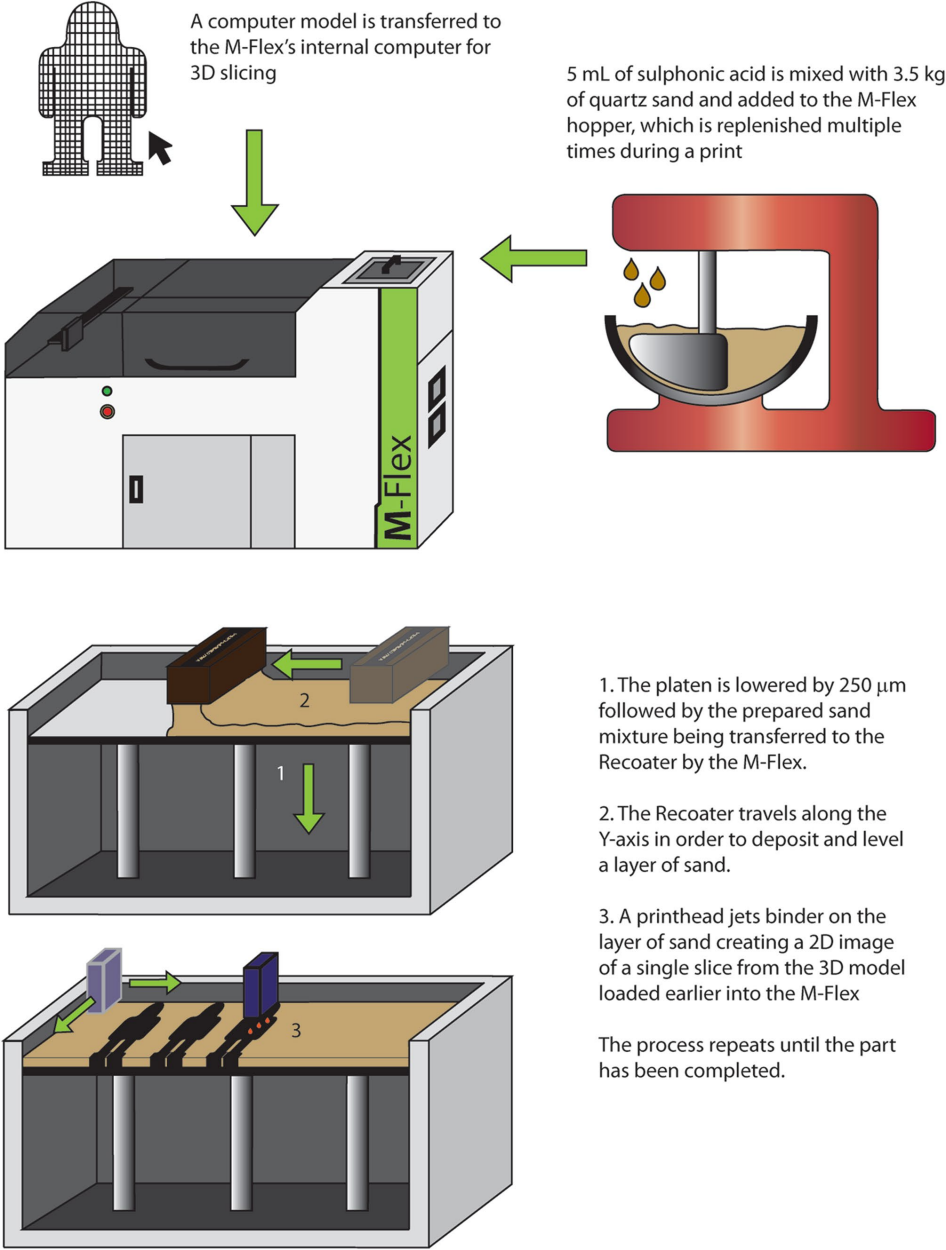
Illustration of the binder jet additive manufacturing process showing the main steps including modelling, preparation of sand media, layering and binder jetting [13]. Reused with permission, Springer © 2018

An M-Flex Sand Printer (ExOne, PA, USA) was used to fabricate the model sandstone in this study. The whole grain silica sand, binder and activator used in the fabrication of the samples was purchased from ExOne. The sand media used was purchased from the manufacturer of the 3D printer (ExOne) and consists of a narrow distribution of particles with a D_50_ of 175 µm, which was obtained via sieving. For printing, 3.5 kg of silica sand (ExOne, PA, USA) was added to a stand mixer (KitchenAid, ON, Canada) followed by the addition of 5 mL of *p*-toluene sulphonic acid (activator) while mixing the sand at low speed for two minutes to coat the sand particles. The acid-coated sand was then added to a hopper at the top of the M-Flex printer. The hopper deposited the sand into a vibrating spreader (Recoater) where upon it was tamped as it moved along the axis parallel to the job box, providing a layer of silica sand approximately 350 µm thick for printing (the job box is a steel container with a platen free to move along the axis normal to the sand layer). The platen drops a specified distance after each layer, allowing a new layer of sand to be deposited by the Recoater. In addition to sand spreading, the print head jets furfuryl alcohol (binder) onto the sand layer while moving along the X and Y-axis, following the pattern of the digital file pre-loaded onto the M-Flex computer. A polymer condensation reaction takes place when the binder comes into contact with the activator on the sand, creating polymer necks between sand grains and solidifying the sand in place. Once the parts were printed, the job box was removed without disturbing the samples and placed into a large oven set to 80 °C where they were left for 24 h. The increased temperature accelerates the curing process and helps reduce moisture in the parts from the condensation reaction during polymerization, which is detrimental to strength [20, 21]. Afterwards, the samples were removed and cleaned with a wire brush to remove any loose sand. An illustration of the printing process can be found below in Fig. 1.

**Fig. 2:**
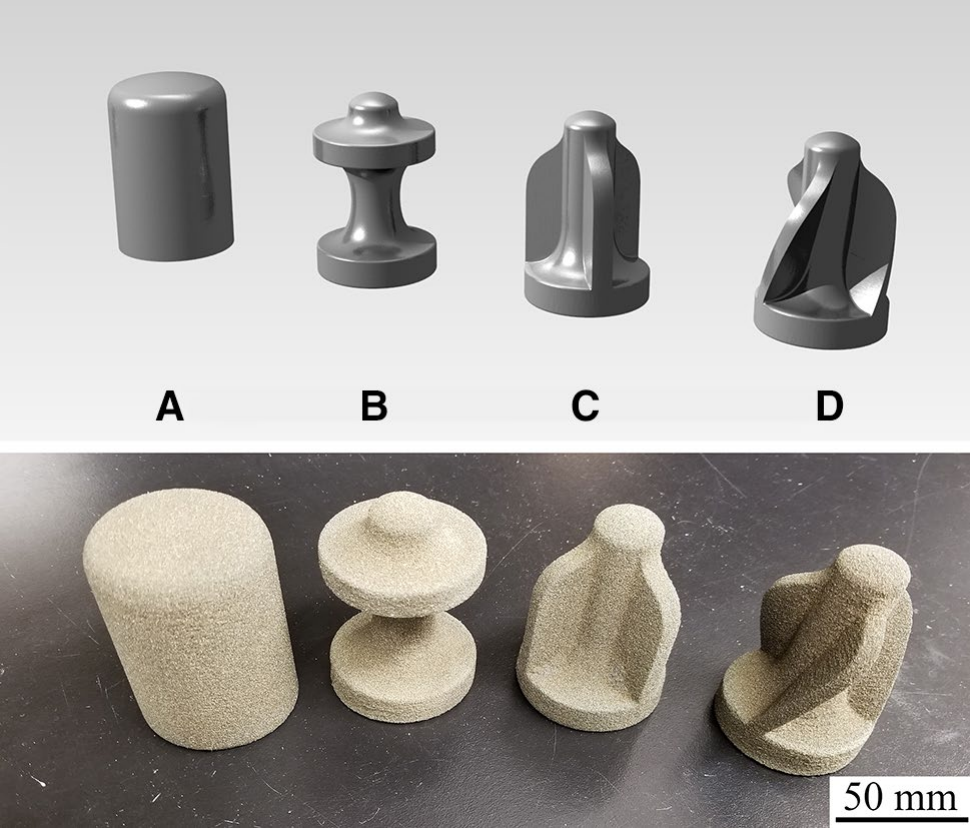
Top: 3D renderings of ballistic designs to be printed for the Royal Military Academy in Belgium, bottom: 3D-printed ballistic samples of various geometries. Samples consist of silica sand bound together with a polymer binder. The designs were chosen to observe the changes in mass and stability

The 3D-printed projectiles were fabricated with set printing parameters, such as layer thickness, travel speed of the Recoater and binder volume fraction. Binder volume fraction is the most important 3D printing parameter as it governs the mechanical strength of 3D-printed specimens [13, 14]. Although not tested directly in this study, the specimens were fabricated with the same printing parameters that resulted in a unconfined compress strength of ~ 20 MPa and a Young’s modulus of ~ 1.8 GPa [16, 22]. The NATO STANdardised AGreement (STANAG) 2920 [23] defines the shapes and masses that should be used for FSPs and is where the basis of our designs began. Four different geometries were fabricated ranging from simple, cylindrical specimens to more complex designs (Fig. 2). Design A is based off the traditional FSP [23], while Designs B, C and D allowed for a significant reduction in mass (~ 50%) compared to Design A. Designs C and D include features for flight stabilization, with Design D incorporating a spiral for the most complex design. The geometries were designed in Rhinoceros 5 followed by exporting to a.STL format that could be communicated to the 3D printer.

### Launching systems

**Fig. 3:**
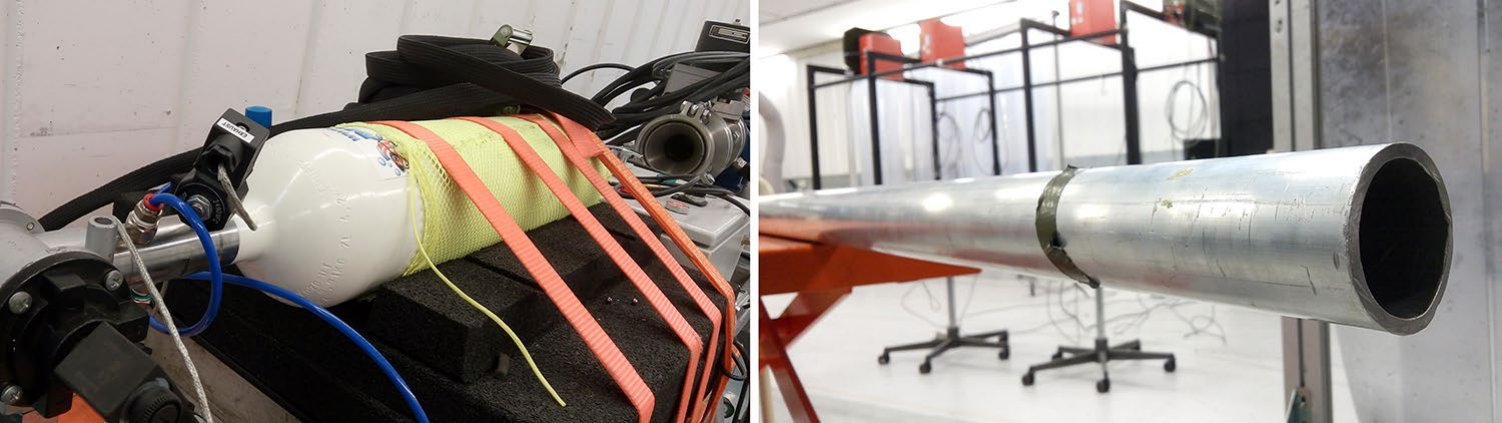
The low-pressure launcher originally used for the first few tests located at the Royal Military Academy in Belgium. The launcher was a pneumatic assembly capable of reaching 0.4 MPa (145 PSI) and included a 1925 mm long and 50 mm diameter barrel

Two pneumatic cannons were used as the launching systems for this study: one for initial observation (low-pressure) and one for terminal ballistic testing (high-pressure). The low-pressure cannon was fabricated with a 10 L pressure vessel customized to fit a 1925 mm barrel having a diameter of 50 mm, including a custom electronic launching circuit and high-speed valve (Fig. 3). The system was capable of reaching a maximum pressure of 0.4 MPa (58 PSI). The second, high-pressure launcher was upgraded to achieve pressures of 7 MPa (1015 PSI) by upgrading the 10 L pressure vessel to 20 L, which allowed for more realistic tests. The same barrel length and diameter was used.

**Fig. 4:**
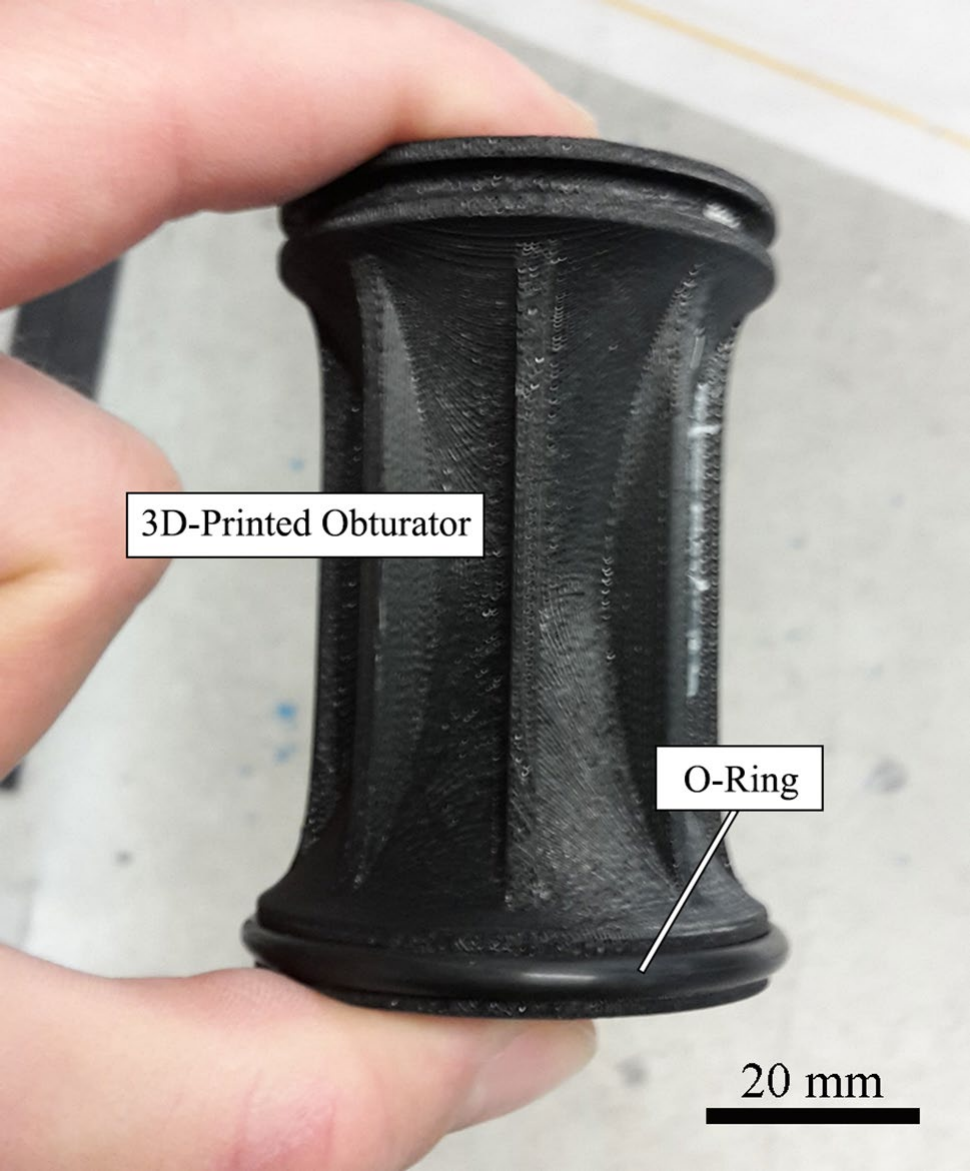
A 3D-printed obturator fabricated from ABS. A slot for an O-ring was included to increase the sealing capability of the obturator since ABS is a rigid plastic

To facilitate launch of the 3D-printed projectiles and reduce air leakage, high density foam obturators were used during launching. In addition, 3D-printed obturators made from ABS (acrylonitrile butadiene styrene) plastic were used to accelerate the samples and provide a seal against pressure loss (Fig. 4). The 3D-printed obturators were designed to fit snugly into the 50 mm diameter barrel and were fabricated on a Fortus 250mc 3D Printer (Stratasys, Israel). A slot was designed into the obturator to allow for an O-ring to be installed to lower air leakage during launch. The last option of reducing air leakage during launch included coating the bottom diameter of the samples in an elastomer coating (Plasti-Dip International, Blaine, MD). Due to the tackiness of the elastomer, oil was applied to the inside of the barrel to allow for a smoother travel down the barrel at launch.

### Projectile testing

All projectiles were captured exiting the launcher using a single high-speed camera system (Photron, SA-2). All velocities and pitch rates were estimated using single frames of the trajectories at discrete intervals. The projectiles were caught via a container lined with soft cloth. Although internal damage to the projectiles from launching was not confirmed, the launching and catching system showed no outward appearance of any significant changes in structure of the 3D-printed projectiles.

For terminal ballistic testing, a single sheet of plain weave Kevlar XP-S102 was attached to a rigid stand. The Kevlar sheet was situated over a box of plasticine (Weible, Germany) to allow for measurements of indentations after testing and provide support against the impact of the projectile. The plasticine was pre-conditioned for a minimum duration of 24 h at 30 °C. The target was placed approximately 1.5 m from the end of the cannon.

## Results and discussion

**Fig. 5:**
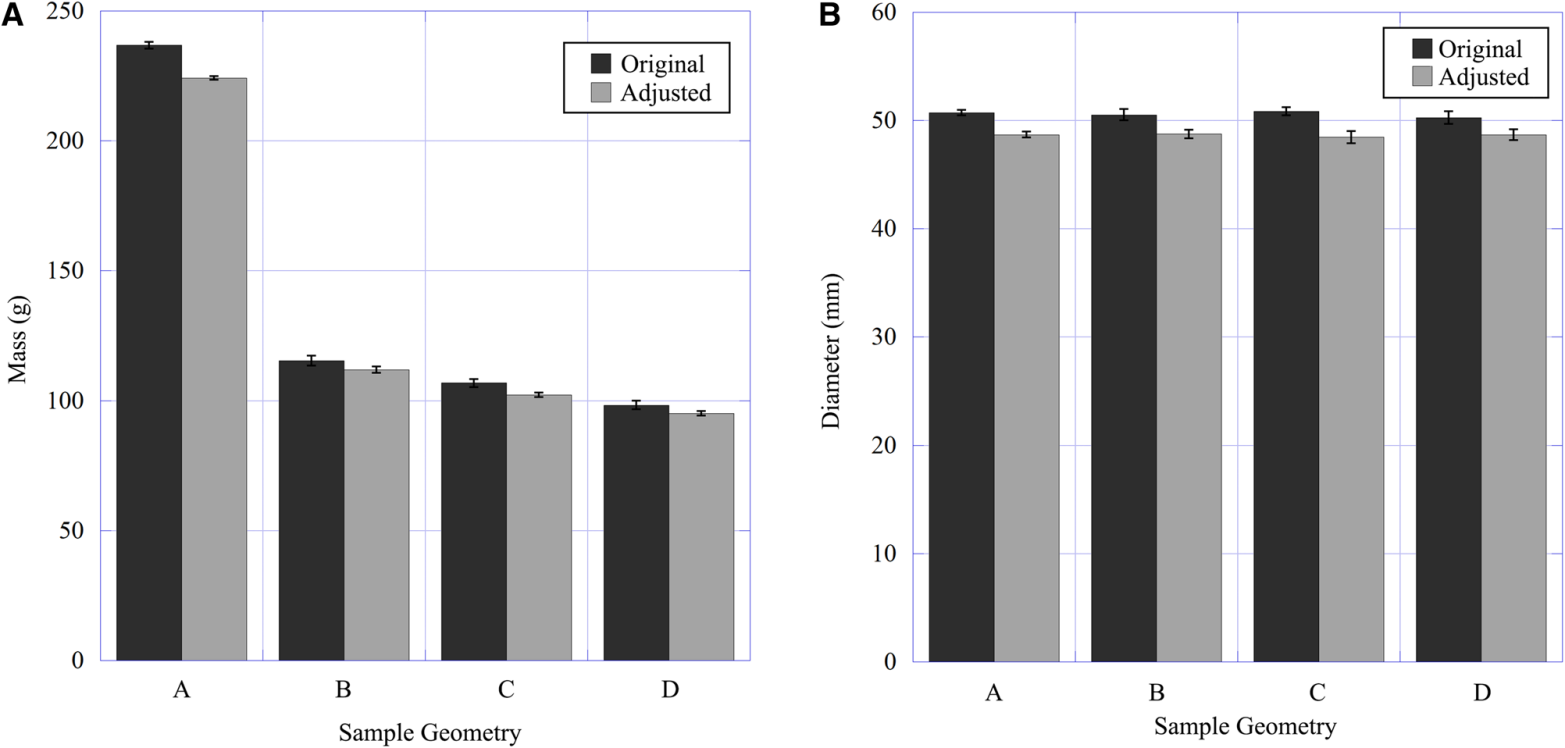
The changes in **A** mass and **B** diameter of the 3D-printed sand ballistics after diameter adjustment via a grinding wheel. Binder bleeding during 3D printing causes the projectiles to have a slightly larger diameter than specified, so the diameter (caliber) must be trimmed for successful launching

The first observation made was regarding the diameter of the projectiles after 3D printing. The actual diameter (caliber) of the projectiles was slightly over the 50 mm diameter stipulated by the digital model, which can be attributed to binder bleeding effects that occur during 3D printing [13]. Binder bleeding is caused by the liquid binder involved in the 3D printing process being drawn into the sand bed through capillary action. The phenomenon causes the two-dimensional layer that is printed on top of the porous sand bed to alter marginally before curing [13]. Although the consequences are minor for most 3D-printed parts, ballistics must maintain accurate dimensions to ensure proper sealing in the barrel. 3D printing provides a fabrication method of producing projectiles with reproducible and accurate diameters, which can be observed through the minimal distribution of both mass and diameter for the as-printed specimens (Fig. 5). A standard rotating grinding wheel was used to manually reduce the diameter to 50 mm to ensure a proper fit in the launcher. The trimmed mass and diameters are compared to the original values in Fig. 5, showing that there was minimal change to both mass and diameter. For all samples, the average mass loss from grinding was 3.9 ± 1.0% (N = 20), while the average diameter loss was 3.8 ± 0.7% (N = 20). By design, projectiles B, C and D had a lower mass than projectile A (50%, 54% and 58%, respectively).

**Fig. 6:**
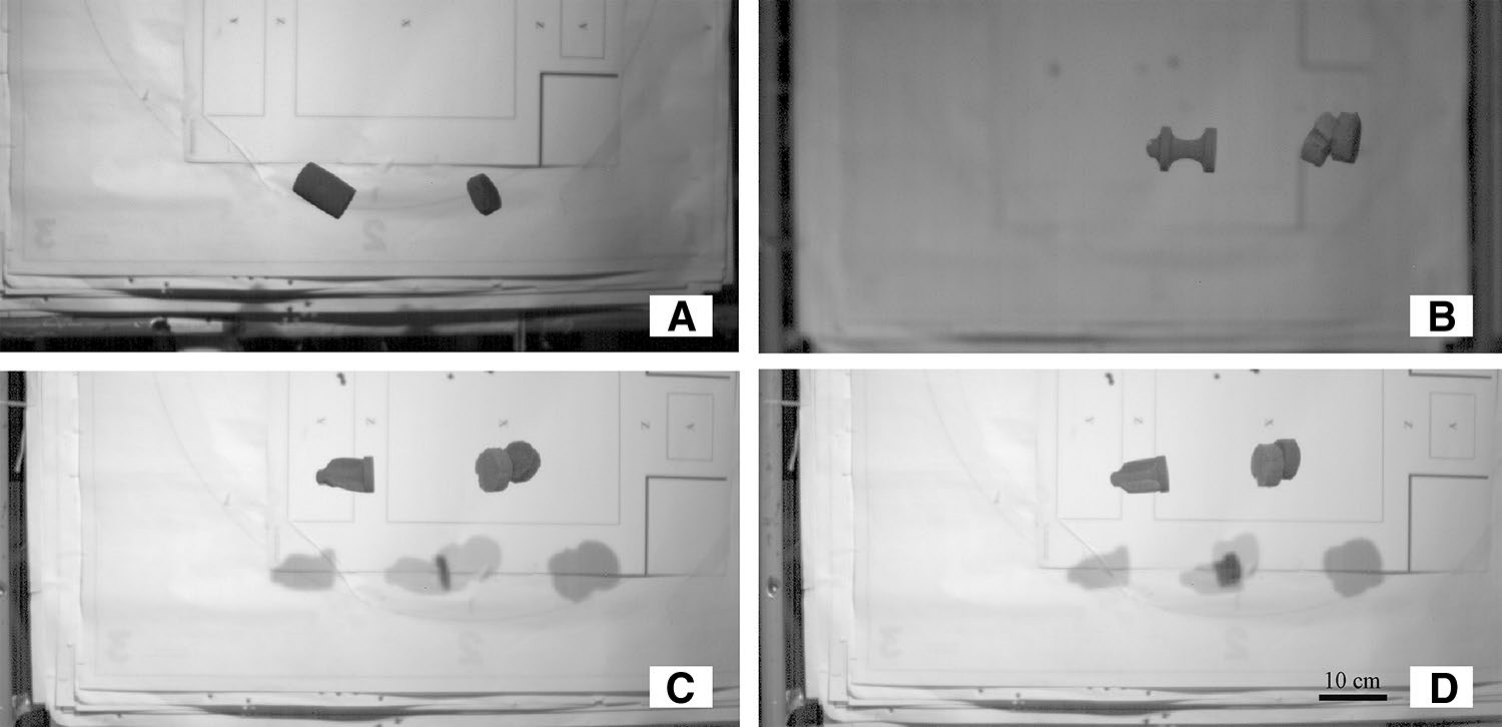
A collage of high-speed photographs, showing in-flight performance of the 3D-printed sand projectiles at low pressure. All projectiles showed instability at low pressures, most likely due to the low velocity

Projectile A was launched at a reduced pressure of 0.3 MPa (44 PSI) in order to assess any premature degradation of the projectiles. However, there was significant destabilization during flight and a low velocity of the projectile was observed. It is suggested that the low velocity and destabilization of projectile A was due to a large amount of air leakage, since a significant pressure drop occurred directly after launch. The high mass/caliber of the projectile also requires significant pressure to achieve viable velocities for fragmentation testing. The sealing issue was addressed by adding high density foam obturators. The maximum pressure for the low-pressure system of 0.4 MPa (58 PSI) was able to launch a single projectile with designs B, C and D for initial observation. When combined with the foam obturators, the velocity of projectile B was increased to 90 m/s. The launch pressures, velocities and pitch rates of the preliminary tests are provided in Table 1. Significant air leaks still accompanied projectiles C and D, which accounts for their lower velocities. Several factors may contribute to reduced velocity such as absence of symmetry (diameters not perfectly round) and high porosity. Due to the sand media and liquid binder used to fabricate the projectiles, the projectiles are rough in texture and perfect axial symmetry is difficult to achieve. The absence of perfect symmetry may account for the significant amount of pitch from all projectiles in Table 1. Additionally, the 3D-printed sand projectiles contain a significant amount of porosity at ~ 43% [16]. The high porosity may allow air to pass around the outer diameter of the projectile, especially if it is out of tolerance. However, the stability of projectile D was improved due to the spiral pattern imposing angular momentum. High-speed photographs of each projectile for the initial testing are shown in Fig. 6. To improve the axial symmetry, future projectiles should be printed with a reduced layer thickness and binder volume fraction, which has been shown to allow greater dimensional control in 3D-printed model sandstone [13, 24].

**Table 1:** The four initial tests of projectile A, B, C and D

Projectile	Pressure (MPa/PSI)	Velocity (m/s)	Pitch Rate (^o^/s)
A	0.3/44	N/A	N/A
B	0.4/58	90	199
C	0.4/58	57	440
D	0.4/58	58	55

Projectile A was launched at a lower pressure to assess any premature degradation, but since the specimen remained intact the remaining three geometries were launched at the maximum pressure of the low-pressure system

### High speed and terminal ballistic testing

**Fig. 7:**
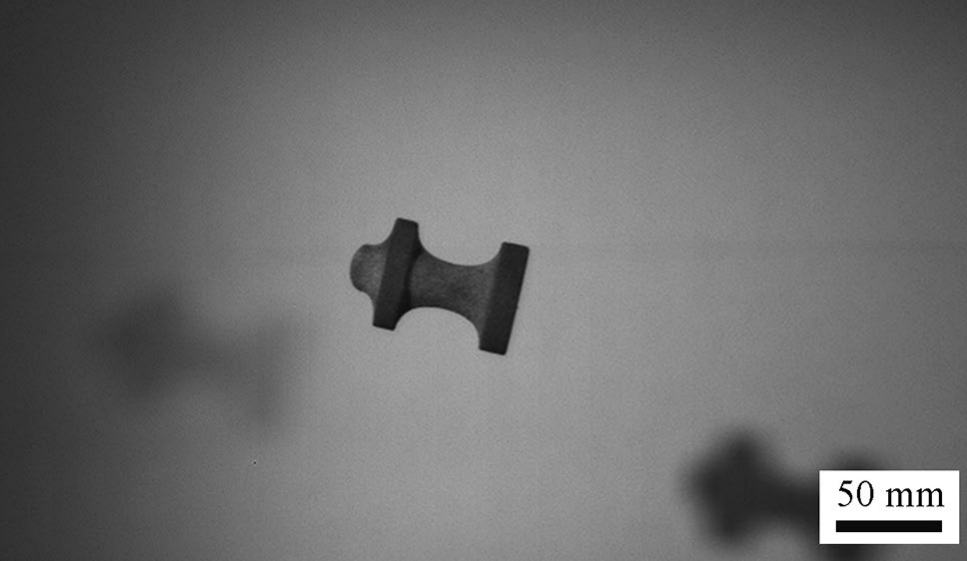
A 3D-printed sand ballistic projectile with a high pitch angle (shooting direction right to left). It is suggested that the deformed foam obturator influenced the unstable flight trajectory

After preliminary testing, the launching system was upgraded to a 20 L pressure vessel. However, it was observed after a few tests that as the pressure increased the velocity did not and in some cases even decreased. It was discovered that the foam obturators underwent extreme deformation during the launches at increased pressure. The foam obturators rotating in the barrel (along with the porous, non-axial symmetric projectiles) would warrant a sufficient pressure drop and increased friction, resulting in the lower velocities observed. The deformed obturator may also have resulted in unstable flight and high pitch angles, which was observed during the initial launches (Fig. 7).

**Fig. 8:**
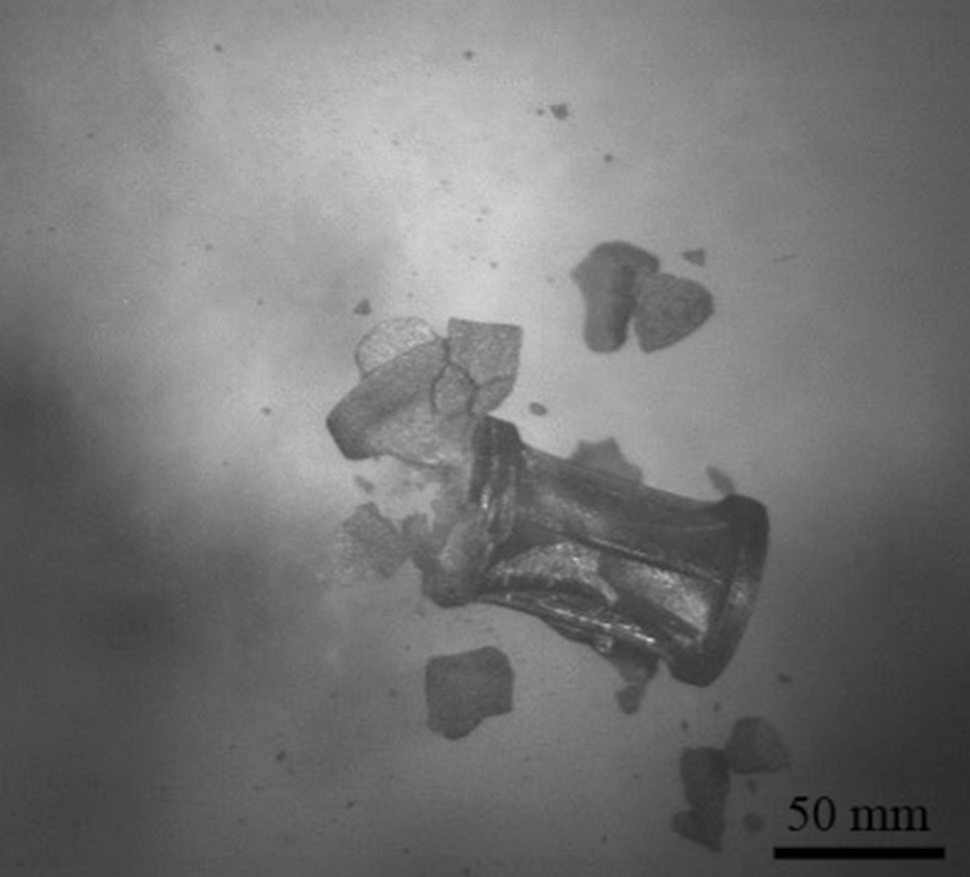
A photograph showing that the sealer used to launch the 3D-printed projectile was too heavy and stiff, causing fracture of the projectile before reaching the intended target

To achieve smoother accelerations and higher velocities, different obturators were tested. The foam obturators were replaced by 3D-printed obturators made from ABS, which showed no evidence of deformation after launching. The 3D-printed obturators allowed for O-rings to be placed at the top and bottom, allowing for a better seal to be achieved (Fig. 4). However, the drawback of a stiff, dense sealer is the increased energy required to accelerate the obturator. Additionally, the kinetic energy on impact with the sample resulted in damage to several projectiles. The 3D-printed obturators trailed the projectile in-flight and did not separate after leaving the barrel. Hence, on impact with the target the obturator impacted the sample from behind leading to fracture. Other times, the obturator fractured the projectile mid-flight (Fig. 8).

**Fig. 9:**
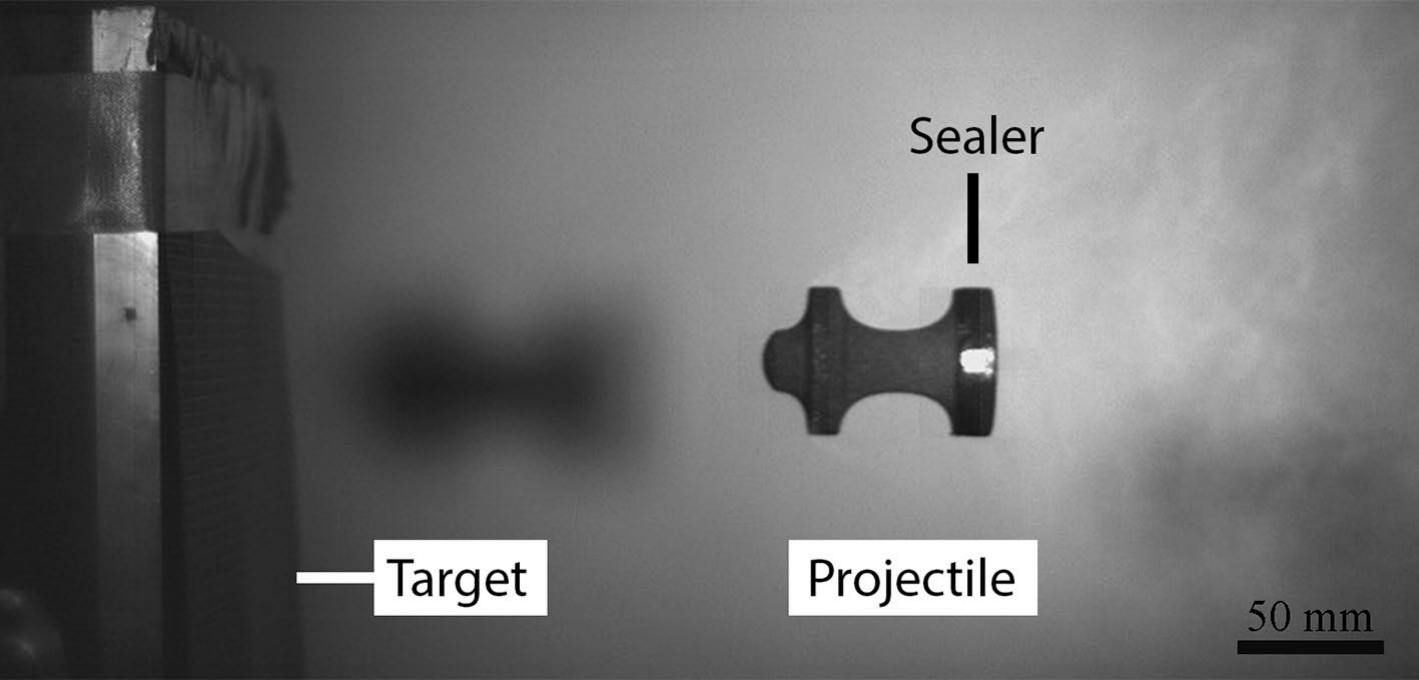
A 3D-printed specimen being launched from the high pressure canon using a new sealing approach, where an elastomer is attached to the bottom of the sample allowing for higher velocities to be reached and more stable flight

**Fig. 10:**
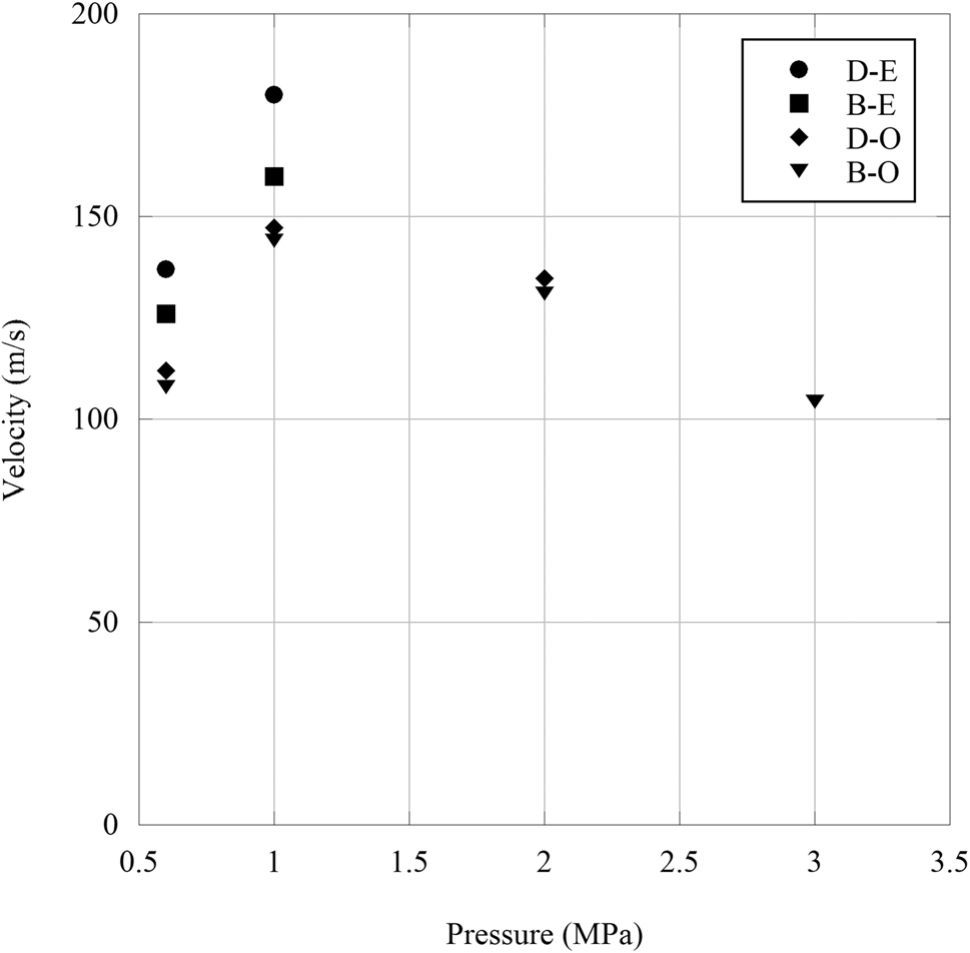
The velocity of different projectiles (B/D = geometric design, E = elastomer and O = 3D-printed obturator). As the air pressure is increased to 1 MPa, the projectiles increase in speed. Past 1 MPa, the projectiles decrease in velocity, which can be attributed to air leakages at higher pressures

The foam and 3D-printed plastic obturators were both problematic, so a decision to coat the projectiles directly with an elastomer was made. By coating the bottom diameter of the projectiles with an elastomeric coating, the projectile was able to maintain a much better seal and achieve a significant velocity gain over the other obturators. The increased velocity resulted in a much more stable flight (Fig. 9). The increase in velocity may be attributed to the reduction of porosity around the trailing edge of the sample. One drawback of this sealing option is that the elastomer coating tends to retain the structure of the 3D-printed sand projectile after impact, which may be undesirable to the initial test conditions of fragmentation. Additionally, due to the induced friction of the elastomer coating, a small amount of oil had to be applied to the inside of the barrel to achieve a smoother launch. Only Samples B and D were selected for coating application and terminal testing due to their speed and pitch rates from previous testing. The changes in mass provided by the coating was minimal at 0.19 ± 0.2% (N = 4). By applying an elastomer coating to the bottom diameter of the 3D-printed sand projectiles, lower pressures were needed to achieve higher velocities than the projectiles launched with a 3D-printed obturator, signifying a more efficient test. The pressures and velocities of all successful launches are provided in Fig. 10.

**Fig. 11:**
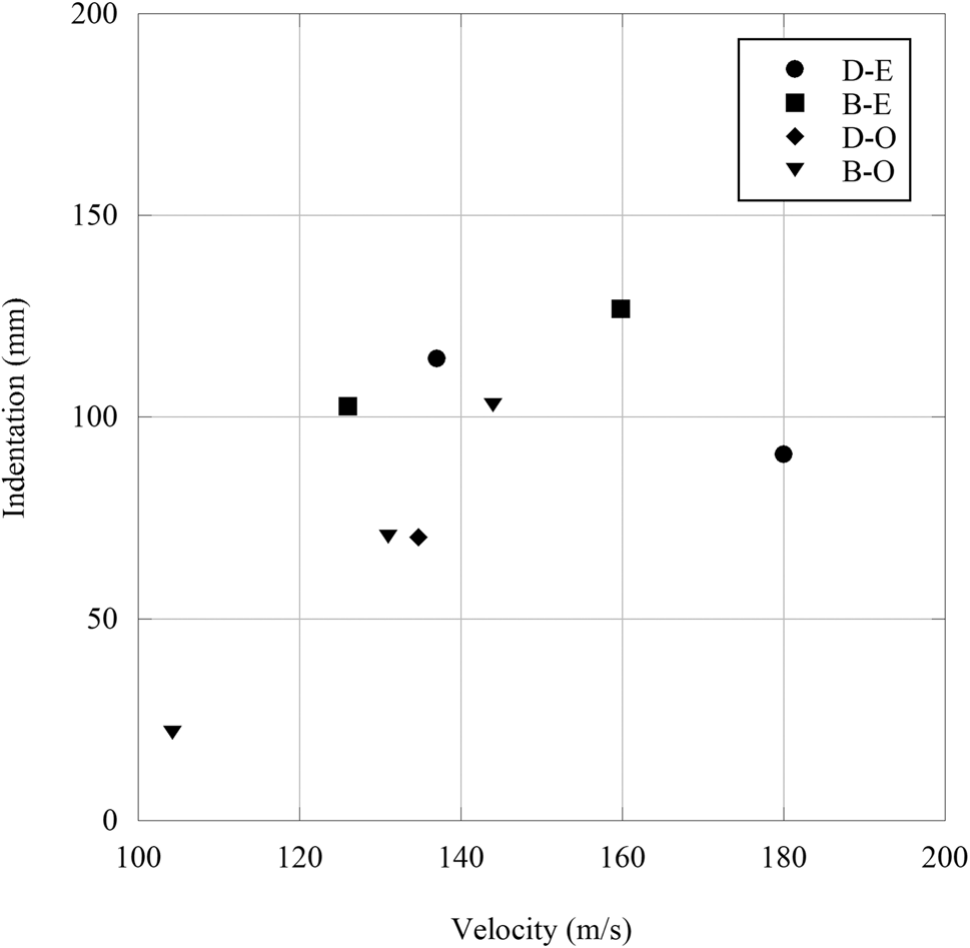
The indentation of different projectiles with respect to their velocity (B/D = geometric design, E = elastomer and O = 3D-printed obturator). There is no correlation between the different types of sealing mechanisms, but as the velocity is increased (increased air pressure) the indentation remains the same, suggesting that an air leak is occurring

**Fig. 12:**
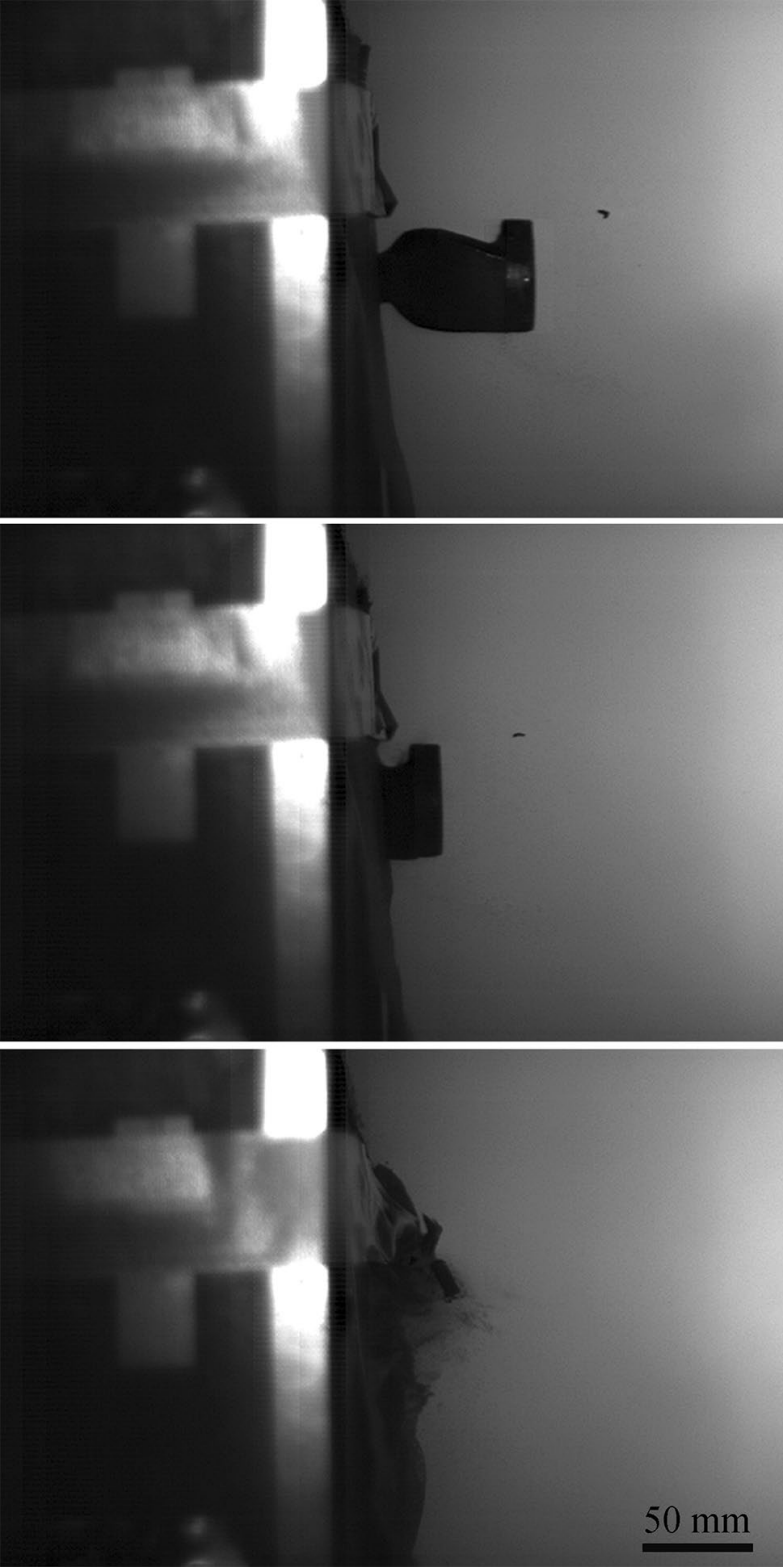
A collage of high speed photographs showing the terminal impact test of 3D-printed sand projectile D. Fracturing of the projectile happened after impact, while the rear of the projectile was held together by the elastomer coating

**Fig. 13:**
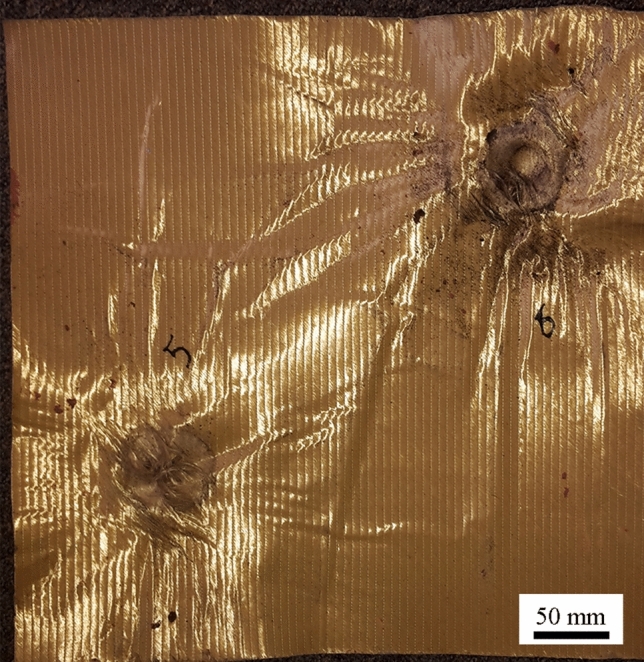
A single layer of Kevlar SP-S102 after being subjected to two terminal ballistic tests. The impact at the top left induced perforation, while the bottom right impact showed no evidence of perforation

In addition to observing flight, terminal ballistic testing was completed to view the impact behaviour of 3D-printed sand projectiles. Projectiles B and D were chosen due to the geometries providing the most stable flights. Projectile A was not used due to the significant mass difference and Projectile C was not used due to the similarity to Projectile D. Both the elastomeric coating and the 3D-printed obturators (to mitigate the effects of the elastomer coating holding the sample together) were used for terminal ballistic testing and the results are shown in Fig. 11. Since the 3D-printed sand ballistics are meant to simulate secondary fragmentation, the ideal target material was an aramid textile. Kevlar XP-S102 was chosen due to its convenient handling and relatively high resistance to Behind Armor Blunt Trauma (BABT) compared to other plain weave textiles. For tests using the ABS obturators, the 3D-printed projectile and obturator traveled as a single body and increased the mass on impact. This introduces error as the obturator is not part of the projectile being tested. Kinetic energy has a significant effect on damage causation in accelerated projectiles [4, 25]. However, the elastomer coating allowed for a projectile that terminated on a sheet of Kevlar as a single object (Fig. 12), where the fracture of the projectile exhibited a brittle response. The debris caused shortly after fracture is in contrast to the plastic deformation and/or perforation normally witnessed with traditional metal FSPs and more closely resembles an accelerated fragmented threat. Yet, due to the caliber and mass of the projectiles, the indentations left in the plasticine were immense. According to NIJ 0101.66 [26], an indentation depth of 44 mm is the upper limit for survivability and all indentations measured within the plasticine were more than twice this amount (example shown by Fig. 13). This damage can be attributed to the size of the projectiles, which would be equivalent to a large rock. Although most of the projectiles penetrated the Kevlar sheet (6 out of 8 launches), due to the limited number of samples any correlations should be taken as qualitative only. Regardless, the large caliber of the projectiles still surpassed the 150 m/s mark as suggested by Freitas [27] for acceptable secondary fragmentation testing, proving that the 3D-printed projectiles show promise at combining natural material into a single projectile that can be launched successfully.

## Conclusions and future work

3D-printed projectiles were fabricated from silica sand and shown to survive launching at speeds required for secondary fragmentation testing. Although significant work needs to be completed on stabilizing the flight of the projectiles, 3D-printed sand ballistics have been shown to be a promising test material for secondary fragmentation damage. It is expected that the velocity may be increased by reducing the caliber and mass of the projectile. Current efforts have reduced the diameter significantly to less than 10 mm, which provide a more accurate assessment of a natural fragment. The axial symmetry can be improved through adjustments to the printing process, which is currently underway.

Moreover, any desired degree of fragmentation could be achieved by implementing cavities and cut-off planes in the internal geometry of the projectiles prior to printing via the digital design, as well as altering the volume fraction of binder within the sample. A higher volume fraction of binder would lead to a stronger projectile, resulting in cracking on impact. A lower binder volume fraction would result in a more friable sample, creating more of a debris of smaller pieces after launch and/or impact. Therefore, the projectiles could possibly be fragmented with the use of an external sharp geometry before hitting the target with minimal loss of kinetic energy. The technology may be extended to vehicle testing or mixtures of ground media specific to a single combat theater, which would open up a new frontier of testing. The promise of 3D-printed sand projectiles for secondary fragmentation testing is a ground breaking technology that warrants further investigation.

## Data Availability

Not applicable. Not applicable.
